# Role of Liquid Composition in the Transient Liquid Assisted Growth of Superconducting YBa_2_Cu_3_O_7‐δ_ Films

**DOI:** 10.1002/adma.202510660

**Published:** 2025-09-09

**Authors:** Lavinia Saltarelli, Diana García, Laia Soler, Elzbieta Pach, Kapil Gupta, Daniel Sanchez‐Rodriguez, Jordi Aguilar, Víctor Fuentes, Eduardo Solano, Cristian Mocuta, Jordi Farjas, Xavier Obradors, Teresa Puig

**Affiliations:** ^1^ Institut de Ciència de Materials de Barcelona ICMAB‐CSIC Campus de la UAB Bellaterra Catalonia 08193 Spain; ^2^ Catalan Institute of Nanoscience and Nanotechnology (ICN2) CSIC and BIST Campus UAB, Bellaterra Barcelona Catalonia 08193 Spain; ^3^ GRMT Department of Physics University of Girona Girona Catalonia 17071 Spain; ^4^ NCD‐SWEET Beamline ALBA Synchrotron Light Source Cerdanyola del Vallès Barcelona 08290 Spain; ^5^ DiffAbs Beamline Synchrotron SOLEIL L'Orme des Merisiers Départementale 128 Saint‐Aubin 91190 France

**Keywords:** chemical solution deposition, epitaxial nucleation, in situ synchrotron X‐ray diffraction, superconducting YBa_2_Cu_3_O_7−𝛿_, transient liquid assisted growth

## Abstract

The unparalleled loss‐less electrical current conduction of high‐temperature superconducting (HTS) materials encourages research on YBa_2_Cu_3_O_7−𝛿_ (YBCO) to unravel opportunities toward numerous applications. Nonetheless, production costs and throughput of the commercialized HTS Coated Conductors (CCs) are still limiting a worldwide spread. Transient liquid assisted growth (TLAG) is a non‐equilibrium process displaying ultrafast growth rate which, when combined with chemical solution deposition (CSD), is emerging as a strong candidate to reduce the cost/performance ratio of YBCO superconductors. This study explores the influence of the (Ba:Cu) molar ratio of the transient liquid composition on the nucleation and growth mechanisms of TLAG. This enables an in‐depth analysis of the critical role of the yttrium supersaturation in the transient liquids considering the out‐of‐equilibrium kinetic character of TLAG. Advanced characterization techniques, including in situ synchrotron X‐ray diffraction, coupled to a multi‐parameter analysis of the contributions to the physical performance, elucidate the influence of transient liquid supersaturation as driving force toward YBCO nucleation and growth. Understanding the fundamental role played by the initial ink composition allows to disentangle how to reach high superconducting performance. The fabrication of high‐performance YBCO films through this novel, high‐throughput growth methodology promotes the use of HTS materials in large scale power applications.

## Graphical abstract

The Y supersaturation in the [Ba‐Cu^(I/II)^‐O] transient liquid composition is the driving force toward YBCO nucleation and growth in TLAG. Tuning the initial (Ba:Cu) molar ratio in the ink composition determines the YBCO epitaxial nucleation through supersaturation control. Highly epitaxial YBCO films derive from ink compositions with high copper content, which finally enable high‐performance superconducting properties.

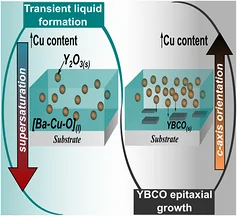

## 1 Introduction

Following decades of worldwide exploitation of fossil fuels, the scientific community is confronted with the compelling need for a global transition toward renewable energy sources. Several solutions have been proposed and the development of novel functional materials with appealing properties has experienced an exponential boost. Cuprate high temperature superconductor (HTS) materials, the best performing being REBa_2_Cu_3_O_7‐δ_ (REBCO, RE = Y or other rare earth elements), opened new possibilities for their implementation in new‐generation devices with unparalleled efficiency of electrical transport and a superior performance compared to the conventional technologies.^[^ ^1^, ^2^, ^3^, ^4^, ^5^ ^]^ Nowadays, HTS are employed in power cables, superconducting magnetic energy‐storage (SMES) devices, transformers, fault current limiters (FCLs), equipment working at ultra‐high magnetic fields (fusion reactors, particle accelerators, NMR),^[^ ^6^, ^7^, ^8^ ^]^ and transport (motors, ships, levitating trains, electrical airplanes).^[^ ^9^, ^10^ ^]^

The high anisotropy of the REBCO perovskite‐like structure demands, however, for the development of manufacturing processes to incorporate these materials in wire fabrication ensuring their biaxial texture with low misalignment at the grain boundaries to prevent loss of supercurrent percolation. Several fabrication methodologies have been employed to produce HTS coated conductors (CCs)^[^ ^4^, ^5^, ^11^, ^12^, ^13^, ^14^, ^15^, ^16^, ^17^, ^18^, ^19^ ^]^ in an industrially feasible process, with the main requirements being the high performance of the REBCO layer contemporarily to the high‐throughput and low production cost to meet the industrial demands. The REBCO layer growth can be achieved through vapor‐solid, solid‐solid, and liquid‐solid growth mechanisms, based on different physical principles. In the three cases, the supersaturation of the system is the driving force for REBCO nucleation and crystallization.^[^ ^4^, ^7^ ^]^ Despite vapor‐solid and solid‐solid growth techniques are the most widely employed in the industrial framework, the slow diffusion kinetics characteristic to these methods limits the growth rate usually below 10 nm s^−1^. Contrarily, the high atomic mobility of liquid‐based methodologies has substantially boosted this parameter, with liquid phase epitaxy (LPE) and its variation, hybrid liquid phase epitaxy (HLPE) demonstrating an increase in the growth rates up to 35 nm s^−1[^ ^20^, ^21^, ^22^ ^]^ or also the reactive co‐evaporation and direct reaction (RCE‐DR) method^[^ ^23^ ^]^ or high‐rate Pulsed Laser Deposition.^[^ ^24^, ^25^, ^26^ ^]^ Liquid‐assisted techniques are promising toward the enhancement of the CCs fabrication throughput, but the use of ultrahigh vacuum methodologies sets a serious challenge for a cost‐wise reduction. Additionally, these techniques operate near thermodynamical equilibrium conditions, limiting the working windows to confined temperature ranges, employing growth temperatures close to 1000 °C and through the disadvantageous use of corrosive melts for timely processes. A breakthrough for feasible low‐cost and high‐throughput CCs fabrication derived from the work conducted in our research group, where the transient liquid assisted growth combined with chemical solution deposition (TLAG‐CSD) was developed and it is now being widely explored.^[^ ^7^, ^27^, ^28^, ^29^, ^30^ ^]^ This novel growth technique relies on the inexpensive CSD methodology^[^ ^31^ ^]^ coupled with the out‐of‐equilibrium growth kinetics of a transient liquid‐assisted process, demonstrating the possibility of unprecedented growth rates beyond 2000 nm s^−1^ in a large window of high epitaxy and compatibility of inclusion of pre‐formed nanoparticles using colloidal solutions.^[^ ^32^ ^]^ TLAG is an out‐of‐equilibrium kinetic growth process driven by the transient liquid supersaturation. Through rapid variations of processing parameters (isobaric thermal variation or isothermal rapid pressure increase, depending on the chosen TLAG growth route^[^ ^28^ ^]^), the formation of a transient liquid of [Ba‐Cu^(I/II)^‐O] is kinetically preferred in a region of the phase diagram where YBCO is the predicted stable phase. Within this liquid remain the Y_2_O_3_ solid nanoparticles. The partial dissolution of the Y_2_O_3_ nanoparticles dispersed in the transient liquid controls the liquid supersaturation and, therefore, the following nucleation and growth rate of YBCO. This liquid‐assisted growth reaction is qualitatively similar to that controlling the formation of bulk YBCO ceramics by melt textured growth^[^ ^33^ ^]^ or thin films by LPE or RCE‐DR, however the real [Ba‐Cu^(I/II)^‐O] liquid composition differs, as well as the temperature – *P_O2_* range where the growth is performed, thus also the nucleation and growth processes.^[^ ^22^, ^23^ ^]^

**Figure 1:**
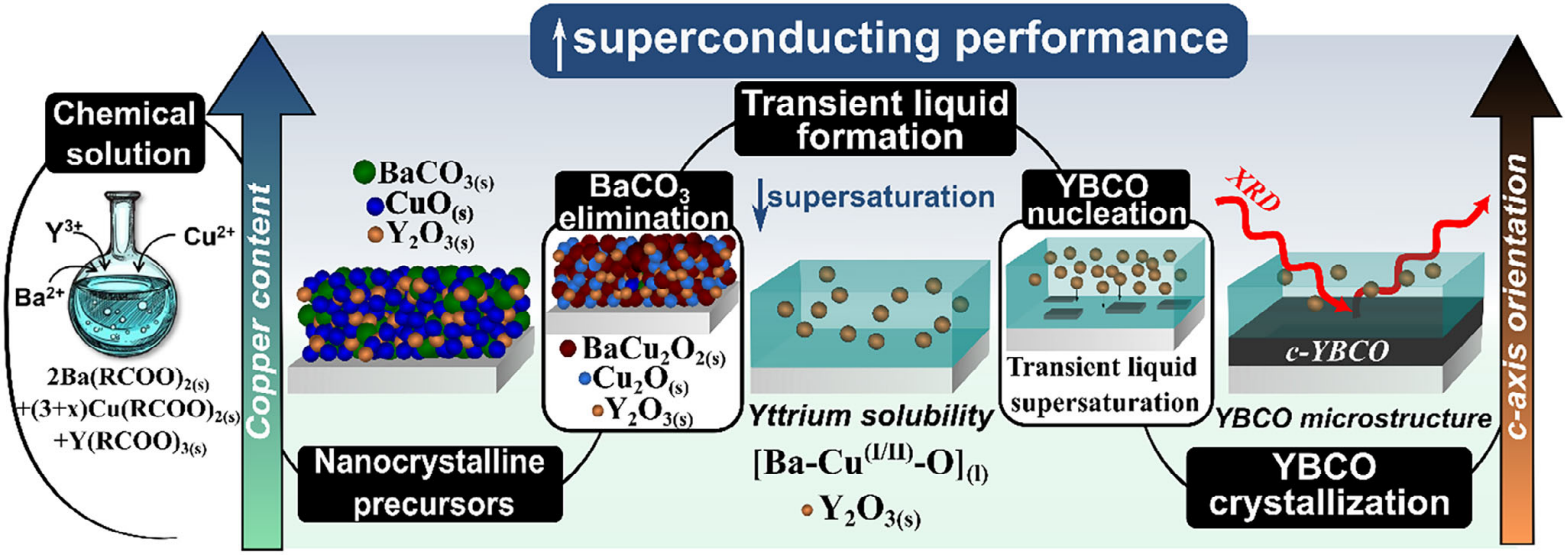
Schematic overview showing the fundamental steps of the TLAG process and how they are correlated. The transient liquid supersaturation, tuned through the initial ink composition, has a direct influence on the final microstructure and superconducting properties. During the TLAG P_O2_‐route, nanocrystalline precursor films composed of BaCO_3_, CuO, and Y_2_O_3_ undergo a low‐P_O2_ annealing where the limiting phase of BaCO_3_ is eliminated upon reaction with Cu_2_O, yielding the mixed oxide BaCu_2_O_2_, Cu_2_O and unreacted Y_2_O_3_, the precursor solid phases for the [Ba‐Cu^(I/II)^‐O] transient liquid formation. Lowering the transient liquid supersaturation through increased copper content of the ink composition allows for higher control of the nucleation process of epitaxial YBCO. In situ synchrotron radiation XRD analysis of the growth of YBCO through TLAG is fundamental to unravel the ultrafast phase transformations and the kinetic implications of the transient liquid supersaturation, to finally enable high‐performance superconducting properties.

The development and optimization of the initial steps of the process, including the preparation of the REBCO chemical solutions, their deposition and pyrolysis to yield oxide‐ and carbonate‐based REBCO precursor films, has been described in previous works.^[^ ^34^, ^35^, ^36^ ^]^ The importance of achieving a nanoscale homogeneity in the pyrolyzed films for the attainment of reproducible high‐performance REBCO films was demonstrated, showing the great potential of TLAG for industrial implementation. Despite kinetic phase diagrams for TLAG identifying regions of phase stability have been previously established,^[^ ^28^ ^]^ a detailed analysis of the role of the transient liquid supersaturation on the epitaxial YBCO nucleation was lacking. In this article, we will focus on the nucleation mechanisms occurring during the ultrafast TLAG kinetic mechanism, presenting the relevant correlations between transient liquid supersaturation and epitaxial nucleation of the final YBCO film and, consequently, on the physical performance. The novelty of the work presented in this article is based on the use of different transient liquid compositions obtained by simple variation of the (Ba:Cu) molar ratio of the initial chemical solution, demonstrating the feasible facile tuning of this parameter. Advanced characterization techniques, such as in situ synchrotron X‐ray diffraction (XRD) of the ultrafast TLAG process, are key to unravelling the influence of transient liquid supersaturation and its successive epitaxial nucleation and growth, generating new and fundamental insights into the complex kinetic mechanism of TLAG. Here, we show that the selection of the adequate liquid composition is a key choice to optimize the growth and critical currents of the YBCO films prepared by TLAG, as summarized in the schematic overview of the entire TLAG process shown in **Figure** **1**.

## 2 Results and Discussion

### 2.1 Y Supersaturation in [Ba‐Cu‐O] Melts

The determination of RE solubility in melts of [Ba‐Cu‐O] has suffered from challenging experimental conditions, given the corrosive nature of the melt, often presenting reactivity with the crucible employed for the analysis. Several of the values reported in literature are strictly dependent on the choice of the crucible material (often showing possible contamination of the melt through reactivity), atmosphere or growth methodology. The concepts and relations provided in this section are, thus, an overview of the general trends of Y solubility in [Ba‐Cu‐O] melts of different composition. However, an analysis of reported values is fundamental to understand the overall tendencies and the further discussion regarding the correlation between transient liquid supersaturation and nucleation in TLAG.

The innovation at the basis of TLAG is the kinetic preference to form a [Ba‐Cu‐O] transient liquid in non‐equilibrium conditions, specifically in regions of the phase diagram where YBCO is the thermodynamically stable phase. Yttrium is found in the liquid as dispersed crystalline Y_2_O_3_ nanoparticles, resulting from the pyrolysis process. The driving force of TLAG is the supersaturation of the transient liquid following the partial Y atomic dissolution from the yttria nanoparticles. The atomic diffusion of the yttrium atoms is enhanced in the liquid, favoring Y migration toward the substrate‐to‐liquid interface which acts as a heterogeneous nucleation site of YBCO nuclei and induces its epitaxial growth, if the processing conditions are selected correctly. In TLAG the energy required for REBCO formation can be described by the difference in chemical potential between the initial transient liquid phase and the final solid REBCO phase (Equation 1). This difference is defined as supersaturation, the driving force for the nucleation process described by Equation (2), in which *C_δ_* is the real RE concentration in the liquid, whereas *C_eq_* defines its concentration in equilibrium conditions. The relative supersaturation, 𝜎, of a melt relates these parameters and is described through the Equation (3).

(1) $$\Delta \mu =\mu_{L}-\mu_{S}$$

(2) $$\Delta \mu =k_{b}Tln\left(\frac{C_{\delta }}{C_{eq}}\right)$$

(3) $$\sigma =\frac{C_{\delta }}{C_{eq}}-1$$

**Figure 2:**
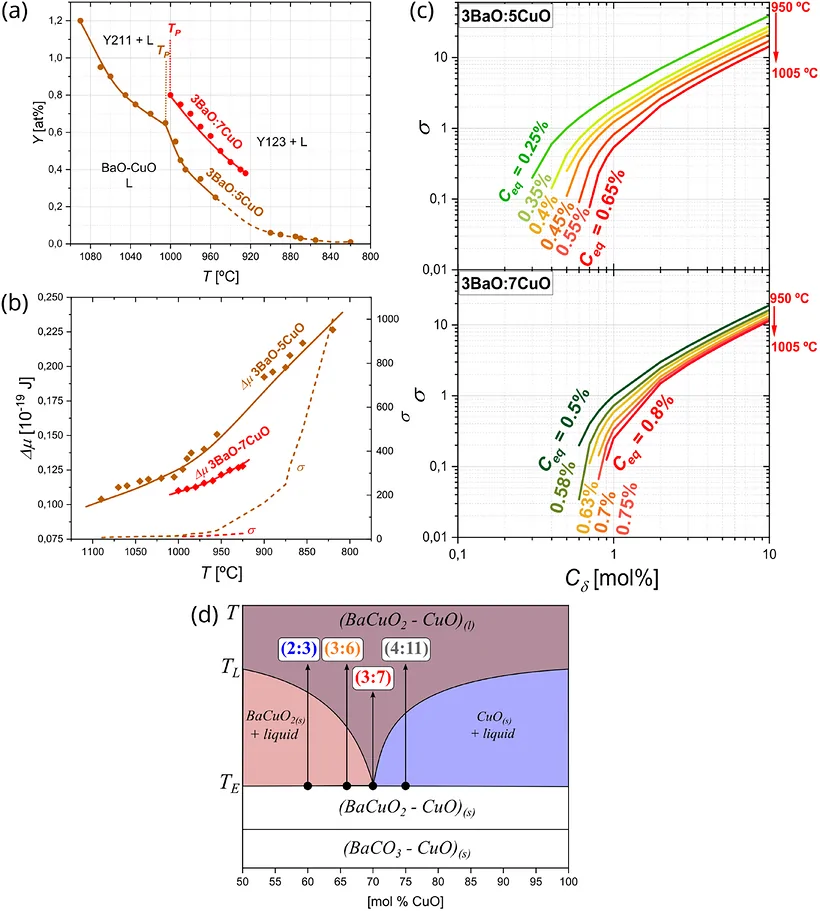
a) *C_eq_* of yttrium as a function of temperature in melts (3BaO:5CuO) and (3BaO:7CuO) compositions (brown and red, respectively). Data points are obtained from,^[^ ^20^, ^37^, ^38^ ^]^ conducted in air. The extrapolation of yttrium solubility to a lower temperature range is obtained from^[^ ^20^ ^]^ exclusively for the former composition and depicted as a dashed line. b) Curves of supersaturation, Δ𝜇 (solid lines), and relative supersaturation, 𝜎 (dashed lines), of yttrium in [Ba‐Cu‐O] melts of (3BaO:5CuO) composition (brown) and (3BaO:7CuO) composition (red). Data points are calculated from Equations (2) and (3), employing values of *C_eq_* in (a) and considering the hypothesis of a fixed *C_δ_* = 10%. c) Calculation of the relative supersaturation 𝜎 of Y in [Ba‐Cu‐O] melts of compositions (3BaO:5CuO) (upper panel) and (3BaO:7CuO) (lower panel), in the temperature range 950–1005 °C with a variation of *C_δ_* between 0.1% and 10%. Corresponding values of *C_eq_* are deriving from the indicated temperature range in (a) and are specified next to the relative curve. d) Schematic representation of the BaCuO_2_‐CuO pseudo‐binary phase diagram adapted from,^[^ ^47^, ^48^ ^]^ to include the liquid compositions described in this work. The validity of this pseudo‐binary phase diagram is restricted to the temperature – *P_O2_* range where the copper ion valence remains Cu^2+^.^[^ ^28^ ^]^ Ratios of Ba and Cu for each liquid composition are specified as (Ba:Cu), corresponding to each mol% of CuO indicated with arrows on the phase diagram. Specific eutectic and liquid formation temperatures are depending on various processing parameters (atmosphere, grain size of precursor phases, sample type). As it is seen in the Figure, in this high *P_O2_* range the low temperature solid phases are BaCuO_2_ – CuO, usually generated from BaCO_3_ – CuO mixtures.^[^ ^28^ ^]^

The driving force toward YBCO crystallization is thus the difference between the real, supersaturated Y concentration in the [Ba‐Cu‐O] transient liquid (*C_δ_*) and its equilibrium concentration (*C_eq_*). Several studies have determined the solubility of Y in equilibrium conditions, *C_eq_*, in melts of two compositions, specifically (3BaO:5CuO) and (3BaO:7CuO), conducted in pure oxygen (*P_O2_* = 1 bar) or in air (*P_O2_* = 0.2 bar) in order to grow bulk ceramics by melt‐textured growth (MTG),^[^ ^37^ ^]^ films by LPE^[^ ^38^ ^]^ and HLPE,^[^ ^21^ ^]^ or single crystals^[^ ^33^, ^39^ ^]^ and the resulting tendency is displayed in **Figure** **2**a. The elevated temperature range is characteristic of these growth methodologies when they are performed in these high *P_O2_* values, but an extrapolation to a lower temperature interval for the (3BaO:5CuO) melt (shown as a dashed line) could be achieved from data in.^[^ ^21^ ^]^ This is a closer approximation to the processing thermal conditions employed for the growth of YBCO through TLAG. Therefore, in the range between 820 °C and 900 °C, *C_eq_* will exhibit values between 0.01 at. % and 0.06 at. %. An overall trend is clearly observed: the composition with higher copper content, here (3BaO:7CuO), displays a generally increased Y solubility in the melt compared to the one with lower copper content, here (3BaO:5CuO), if considering the same temperature range. These findings have been at the origin of the use of melt compositions with copper excess for YBa_2_Cu_3_O_7‐_ *_δ_* formation. If considering the case of one composition exclusively, one may promote the Y solubility in that melt through a simple temperature increase. Figure 2a is thus giving an estimation of the equilibrium concentration, *C_eq_*, of Y in melts of two compositions as a function of the temperature. It is a complex matter to estimate the value of the real Y concentration *C_δ_* and this objective has not been fulfilled up to now. However, an estimated value (*C_δ_* ≈ 10%) can be obtained from an extrapolation to low temperature of the Y_2_O_3_+L liquidus line of the equilibrium phase diagram of the (3BaO:5CuO) liquid (Figure S1, Supporting Information). Therefore, using this value of *C_δ_*, the relation between supersaturation, Δ𝜇, and consequently relative supersaturation, 𝜎, with the temperature can be estimated under this hypothesis. The computed curves for both parameters for the (3BaO:5CuO) and the (3BaO:7CuO) liquids (assuming that *C_δ_* ≈ 10% is also valid here) are shown in Figure 2b. It is evident that Δ𝜇, and the directly related 𝜎, follow an indirect correlation as the one displayed in Figure 2a, and are both lowered with the increase of the copper content of the specific liquid composition analyzed. Nucleation may be, thus, favored when considering the same thermal conditions for a composition with lower copper content, i.e., the strategy to promote nucleation at a constant temperature is to increase the concentration of the solute (Y solubility in the liquid). A higher supersaturation will increase the nucleation rate.^[^ ^40^ ^]^ However, several authors^[^ ^41^, ^42^ ^]^ have reported the direct correlation between elevated supersaturation and an enhanced nucleation rate of unfavorable orientation of YBCO grains (a‐axis heterogeneous epitaxy and homogeneous nucleation of randomly oriented grains), undesirable for the application proposed in this work. Considering the same temperature range (950‐1005 °C, corresponding to the peritectic temperature of YBCO determined in these studies conducted in air) and the values of *C_eq_* deriving from Figure 2b, and taking into account the uncertainty about the real *C_δ_* values, the calculation of 𝜎 for each value of *C_eq_* has been performed as a function of the variation of *C_δ_* (between 0.1% and 10%). The resulting curves of 𝜎 in each of the two [Ba‐Cu‐O] melts considered, (3BaO:5CuO) and (3BaO:7CuO), are displayed in Figure 2c, respectively upper and lower panels. At the lowest temperature (950 °C), *C_eq_*, thus Y solubility, is doubled in the melt of higher copper content, leading to a maximum value of 𝜎 of 19 (when *C_δ_* = 10% is selected). Contrarily, the relative supersaturation of Y in the melt of reduced copper content reaches a maximum value of 39 in the same conditions, over a factor 2 higher. In general, the same trend is observed with the increase of temperature, as it is evident from the elevated values of *C_eq_* exhibited by Y in the former melt. Nonetheless, with the aim of reducing the relative supersaturation inside the same system, the temperature can be increased to promote Y solubility. At the present stage, it can't be discarded from our theoretical analysis that *C_δ_* differs for different liquid compositions; however, it is unlikely that a reduced supersaturation in the liquids with higher copper content is reached. The experimental investigations reported later confirm this expectation.

Further reports are additionally focused on understanding the melting mechanism in BaO‐CuO mixtures with the aim of controlling YBCO nucleation from such melts.^[^ ^43^, ^44^, ^45^, ^46^ ^]^ The effort of all these studies has led to the construction of a pseudo‐binary phase diagram of the BaCuO_2_‐CuO system through the analysis of quenches of said mixtures (at *P_TOT_* = 1 bar) in a variety of gas atmospheres. A schematic model of this (Figure 2d) will aid us in defining the transient liquid compositions employed in this work, as the mol% of CuO of the four liquid compositions considered is clearly indicated.

As it may be observed, the low temperature solid phases in the temperature – *P_O2_* region of the phase diagram where copper has the Cu^2+^ valence are the mixture (BaCuO_2_ – CuO)_s_, deriving from the decomposition of BaCO_3_, which is then the limiting step for the formation of BaCuO_2_. In the temperature – *P_O2_* range of the phase diagram where Cu^+^ is stable the corresponding solid phases are BaCu_2_O_2_ and Cu_2_O and the Cu_2_O – BaCO_3_ reaction is the limiting step to advance in the formation of the transient liquid precursors.^[^ ^28^ ^]^ *T_E_* is indicating the eutectic line, determining the melting onset temperature. The (3:7) composition is the sole to show congruent melting at the eutectic point, where the (Ba:Cu) ratio corresponds to 3(BaO):7(CuO). For compositions with lower copper content, specifically the stoichiometric (2:3) composition and the first investigated with copper excess respect to that, the (3:6) composition, the BaO‐CuO mixture melts partially and displays the presence of crystalline BaCuO_2_, until the liquidus temperature, *T_L_*. Contrarily, the (4:11) composition shows the presence of solid CuO after partial melting and the increase of Cu content in the liquid tends to enhance the solubility of the RE ions.^[^ ^49^ ^]^ Above *T_L_* in all cases, the homogeneous melts of BaCuO_2_‐CuO with different (Ba:Cu) ratios are generated. Therefore, we explored the use of compositions with great copper excess with the aim of lowering the supersaturation intrinsically in the transient liquid composition, with the final objective of promoting the c‐axis nucleation in the system.^[^ ^41^, ^42^ ^]^ Moreover, this would result in a further approach to reduce the optimal processing temperature for c‐axis YBCO growth, particularly interesting when considering the feasibility of TLAG in industrial processes of higher energetic efficiency. A variation of the (4:11) composition, namely Y‐rich (4:11) composition, is additionally employed (Y:Ba:Cu being 1.35:2:5.5) to clarify the influence of Y_2_O_3_ excess in the TLAG process.

### 2.2 Influence of Film Composition on Transient Liquid Formation and Epitaxial Nucleation

In the TLAG P_O2_‐route employed in this work,^[^ ^28^ ^]^ the formation of BaCu_2_O_2_ at low *P_O2_* values (*P_O2_* ≈10^−5^ bar) is crucial for the advancement of the growth process when an isothermal *P_O2_* jump is performed. A complete formation of the [Ba‐Cu^(I/II)^‐O] transient liquid (i.e., a liquid with selected different Ba‐Cu ratios and a mixed Cu^+^ – Cu^2+^ valence which depends on the selected temperature and *P_O2_* values) derives from the melting of the two solid precursor phases (BaCu_2_O_2_ and Cu_2_O), resulting from the reaction of BaCO_3_ and Cu_2_O during the low‐*P_O2_* heating.

A detailed study of the limiting step of the process, the elimination of BaCO_3_, demonstrated that films with lower copper content (namely, (2:3) and (3:6)) present complications to achieve full BaCO_3_ elimination, likely attributable to characteristics of the film microstructure. Contrarily, higher copper contents ((3:7) and (4:11)) lead to a complete and straightforward elimination of this phase, showing the relevance of microstructural and compositional features in the BaCO_3_ removal. Please refer to SI, Section II for a detailed explanation of this analysis.

**Figure 3:**
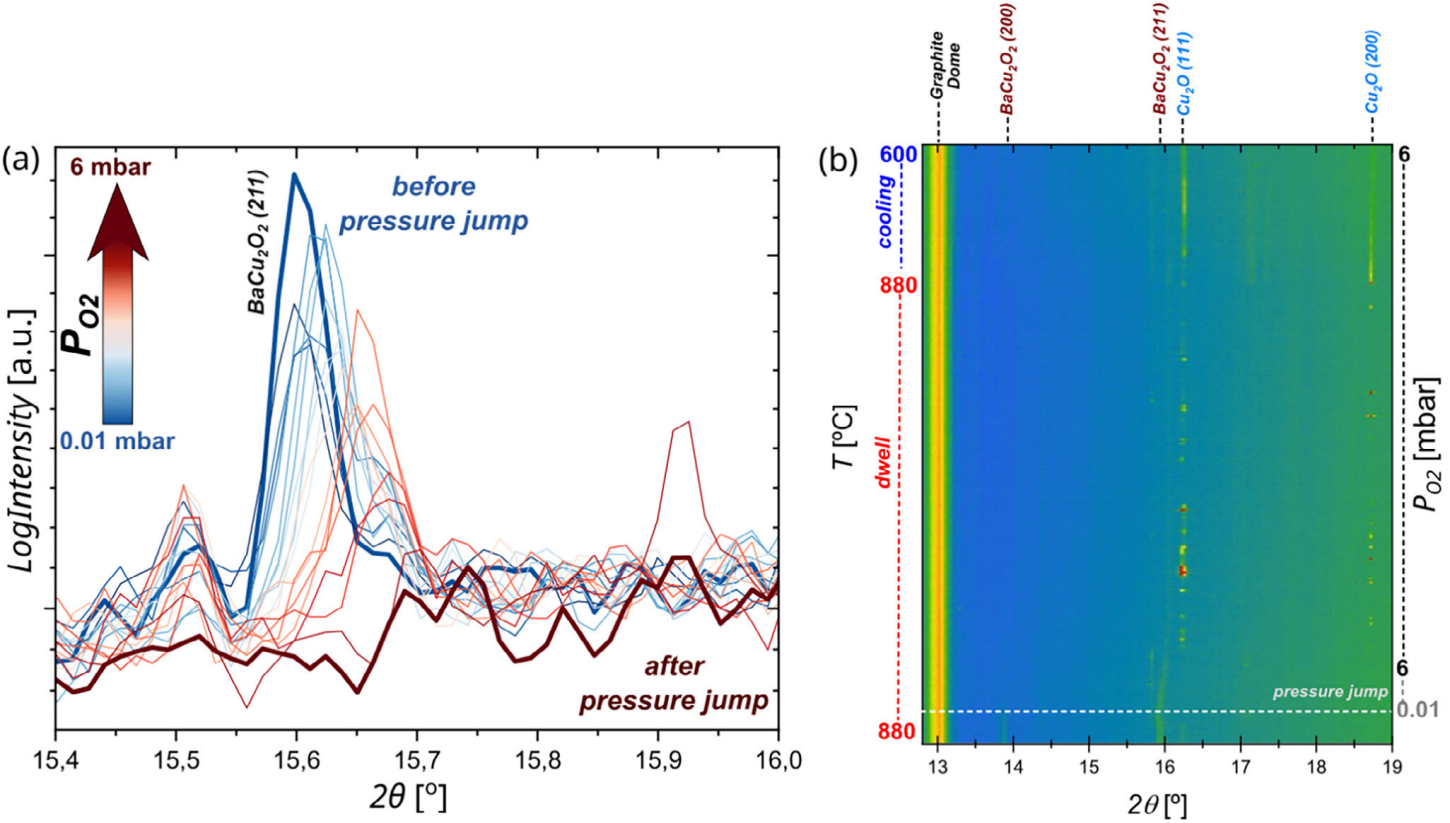
a) *I(2θ)* profiles (displayed with intervals of 10 profiles) resulting from the integration in 𝜒 of the 2D‐XRD images acquired during the pressure jump of a ternary Ba‐Cu‐O film of eutectic composition at *T* = 880 °C, from *P_O2_* = 0.01 mbar to *P_O2_* = 6 mbar, thus in the full liquid region of [Ba‐Cu^I^‐O]. Focus is set on the *2θ* range where the BaCu_2_O_2_ (211) reflection appear, showing no presence of peaks relative to this phase after the jump. The profiles were acquired at ALBA synchrotron with X‐Ray energy of 18 keV. b) Time‐dependent phase evolution color map relative to the sample shown in (a) before the *P_O2_* jump, during an isothermal dwell and during cooling. Crystalline phases are identified according to the *2θ* position of the Bragg reflections on the upper part of the graph. Temperature and pressure conditions are indicated as y‐axes for a comprehensive visualization. The starting frame relative to the pressure jump is indicated on the graph as a grey dashed line.

It is crucial to understand the behavior of the transient liquids formed at the low *P_O2_* values used in the TLAG process, therefore, we first investigated the melting process of TLAG using a Ba‐Cu‐O ternary film, with the (Ba:Cu) molar ratio corresponding to that of the eutectic (3:7) composition. Excluding Y from the composition will impede YBCO crystallization and allow us to determine the phase transformations from the solid phases of BaCu_2_O_2_ + Cu_2_O to the full liquid of [Ba‐Cu^I^‐O], expected for the selected final processing conditions of *T* = 880 °C and *P_O2_* = 6 mbar (*P_TOT_* = 35 mbar). **Figure** **3**a displays the *I(2θ)* profiles regarding the *2θ* range where the principal BaCu_2_O_2_ Bragg reflections appear during the change in *P_O2_*, whereas Figure 3b shows the time‐dependent phase evolution as a color map depicting the intensity of the analyzed crystalline phases. The film is heated first at low *P_O2_* = 0.01 mbar until 880 °C. Here, the presence of crystalline contributions of BaCu_2_O_2_ and Cu_2_O prior to the jump are resulting from the elimination of BaCO_3_ through its reaction with CuO. These are the solid phases expected at these conditions and required for a straightforward formation of the transient liquid. During the *P_O2_* jump, the intensity of the crystalline contributions decreases, with no BaCu_2_O_2_ Bragg reflections present after the jump (as visible in Figure 3a). The film is then maintained for several minutes in a dwell at *T* = 880 °C and *P_O2_* = 6 mbar (Figure 3b). The absence of any continuous crystalline contribution relative to BaCu_2_O_2_ suggest the successful formation of a transient liquid of [Ba‐Cu^I^‐O] is occurring during the dwell. The observed minute discontinuous variations of the weak Bragg reflections ((111) and (200)) are not correlated and so, very likely, these peaks correspond to grain reorientations of remaining Cu_2_O solid particles within the liquid. When performing initial cooling of the film, crystallization of Cu_2_O occurs.

**Figure 4:**
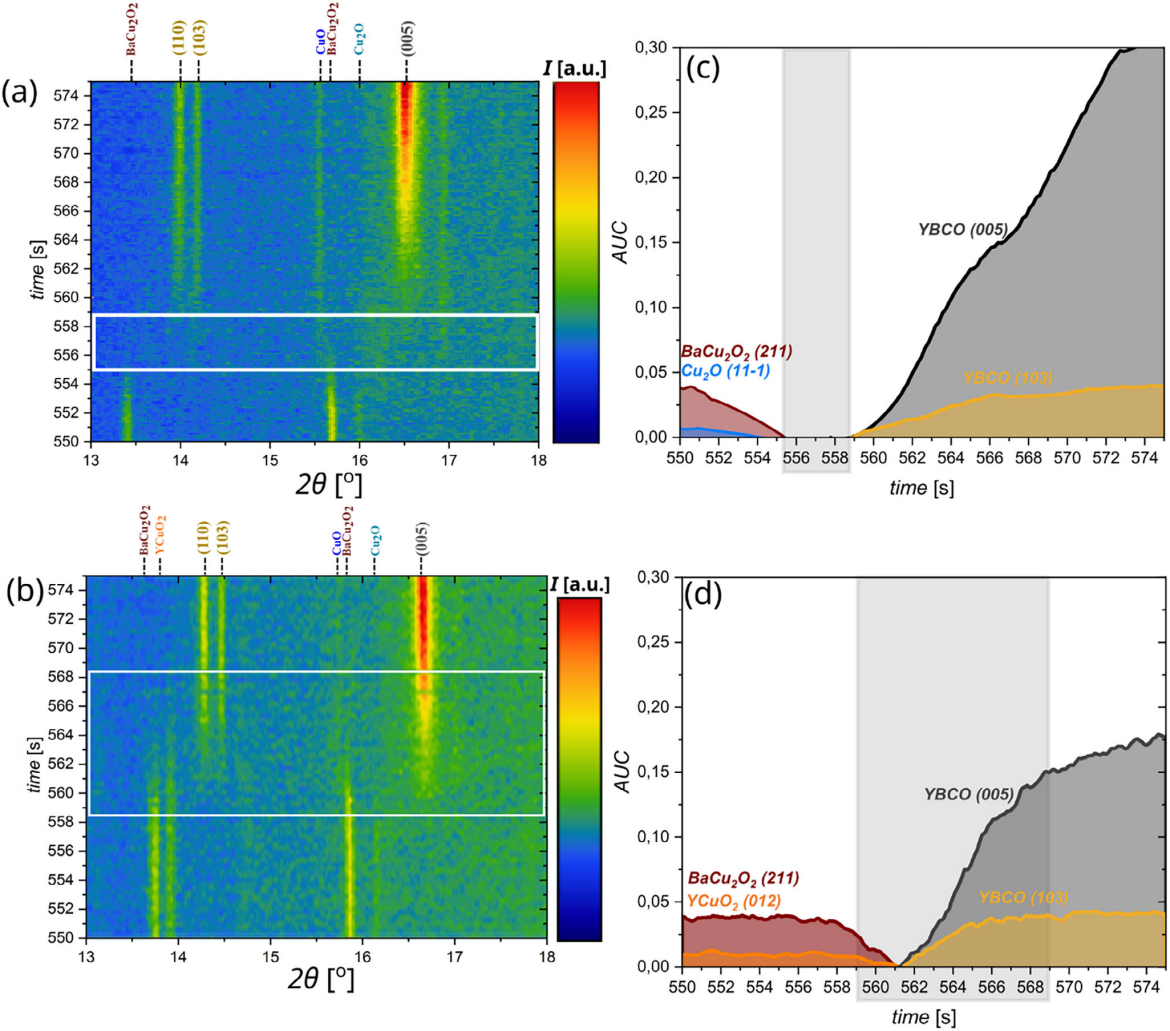
Time‐dependent phase evolution color map showing the time range corresponding to the pressure jump (*T* = 850 °C, from a *P_O2_* = 0.01 mbar to *P_O2_* = 7 mbar) for a) a quaternary Y‐Ba‐Cu‐O (4:11) composition film and b) a quaternary Y‐Ba‐Cu‐O (2:3) composition film grown through TLAG. Identification of the phases according to their *2θ* peak positions is specified in the upper part of the graph. c,d) Time‐dependent phase evolution plots (deriving from the integration of the peaks relative to the crystalline phases), with the grey shading evidencing the same time range identified by the grey rectangle corresponding to plots in (a) and (b). The profiles were acquired at SOLEIL synchrotron with X‐Ray energy of 18 keV.

When including Y propionate in the starting solution, the partial dissolution of the dispersed Y_2_O_3_ nanoparticles formed after the pyrolysis in the transient liquid will rapidly vary its supersaturation, which will drive the ultrafast liquid‐assisted YBCO nucleation and crystallization. Conducting the phase evolution analysis on quaternary Y‐Ba‐Cu‐O films, we identify that microstructural and compositional features of the films prior to the pressure variation are influencing the formation of the transient liquid for films grown in the full‐liquid region of the kinetic phase diagram. In the case of the eutectic and the highest copper excess compositions, the straightforward elimination of the limiting phase of barium carbonate during the low‐*P_O2_* heating leads to films with sole presence of the two precursor phases for the transient liquid formation, BaCu_2_O_2_ + Cu_2_O. When performing the *P_O2_* jump, these phases immediately lead to a complete transient liquid formation. The time‐dependent phase evolution plots displayed in **Figure** **4**a following the evolution of the crystalline phases during the *P_O2_* jump in the particular case of a film of (4:11) composition is demonstrating that the film lacks any crystalline phase in a time range of 3.8 s following the disappearance of the BaCu_2_O_2_ and Cu_2_O reflections. This is a strong evidence of the complete formation of a [Ba‐Cu‐O] transient liquid from which the crystallization of YBCO is occurring. Analyzing the same process for a film of the stoichiometric (2:3) composition, we found that crystalline contributions are always present, with a slight overlapping of the disappearing BaCu_2_O_2_ reflections and the new reflections of YBCO associated to crystallization. It is very likely that some remaining BaCO_3_ in the film prior to the *P_O2_* jump delays the phase transformation to BaCu_2_O_2_, consequently retarding the formation of a full transient liquid across the entire film. Enabling the formation of a full transient liquid strongly favors the achievement of highly epitaxial films which are demonstrated to grow with unprecedented growth rates of 2000 nms^−1^.^[^ ^28^ ^]^

**Figure 5:**
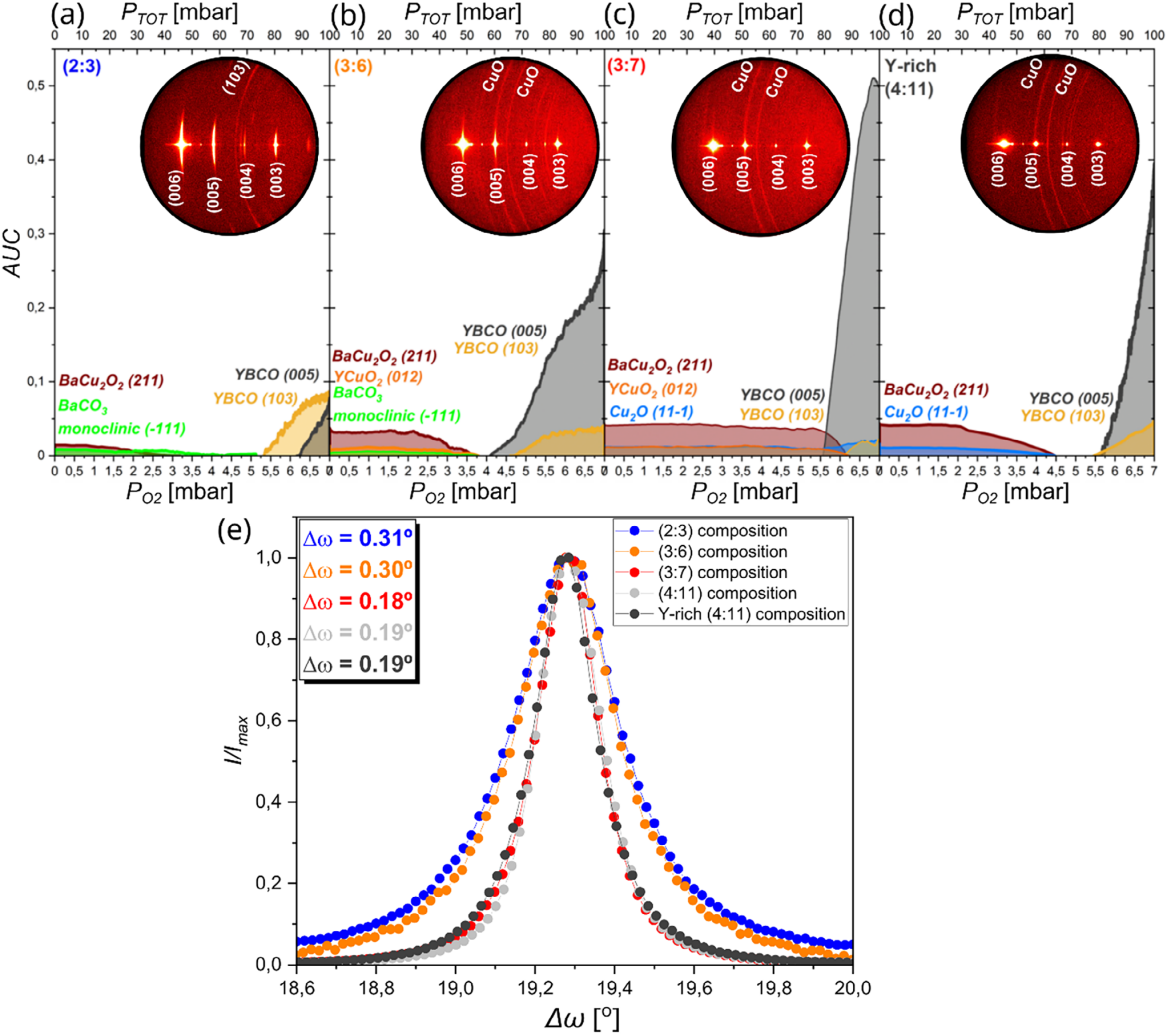
Pressure‐dependent phase evolution plots relative to the pressure jump of films of a) (2:3), b) (3:6), c) (3:7), and d) (4:11) liquid compositions acquired through in situ synchrotron XRD (*T* = 850 °C, from a *P_O2_* = 0.01 mbar to *P_O2_* = 7 mbar and *P_TOT_* from 0.05 to 100 mbar), with the respective ex situ 2D‐XRD image shown as inset (acquired with X‐Ray energy of 8.049 keV). Identification of the crystalline phases is shown in each plot and in the 2D‐XRD image for clarification. In the phase evolution plots, raw peak area is displayed with the same scale for all plots to evince differences on epitaxy due to each liquid composition. e) Rocking curves (ω‐scans) corresponding to the films shown in (a)‐(d), with specific *Δω* values indicated in inset.

The effect of the initial film composition on both the process of elimination of BaCO_3_ and on the formation of the transient liquid is consequently influencing the epitaxial nucleation of the final YBCO film. The evolution of phases observed through in situ XRD is analyzed to compare the growth of films of the four liquid compositions considered in this work. Performing TLAG in the same processing conditions allows to evidence the strict correlation between supersaturation and c‐axis YBCO nucleation. The evolution of phases with *P_O2_* shown in **Figure** **5** evidence the more favorable conditions of the (3:7) and (4:11) compositions (panels (c) and (d), respectively) to generate predominantly c‐axis orientation of the crystallized YBCO films. From the corresponding 2D‐XRD images (inset), the eutectic composition is discerning between optimal c‐axis nucleation conditions, with the two compositions with higher copper excess displaying similarly high quality epitaxy (intense diffraction spot attributable to the YBCO *(00l)* reflections together with the rings due to polycrystalline CuO, deriving from the copper excess in these compositions). This is in contrast to the low‐copper content films which exhibit a strong misorientation around the c‐axis (identifiable as rings around the *(00l)* diffraction spots). The mixed nucleation of misoriented and c‐axis YBCO is also clear in the respective pressure‐dependent phase evolution plots (Figure 5a,b), where in the case of the stoichiometric (2:3) composition, these phases reach similar intensity values. In addition, Figure 5e shows comparison of the rocking curves (ω‐scans) of the films in Figure 5a–d. The correlation between elevated copper content of the liquid composition and high epitaxial texture of the film is again evident, with the compositions of higher copper content (namely, the eutectic and the (4:11) composition) yielding extremely low *Δω* values down to 0.18°. Contrarily, rocking curves of compositions with lower copper content ((2:3) and (3:6) compositions) display similarly broader FWHM around *Δω* = 0.30°.

**Figure 6:**
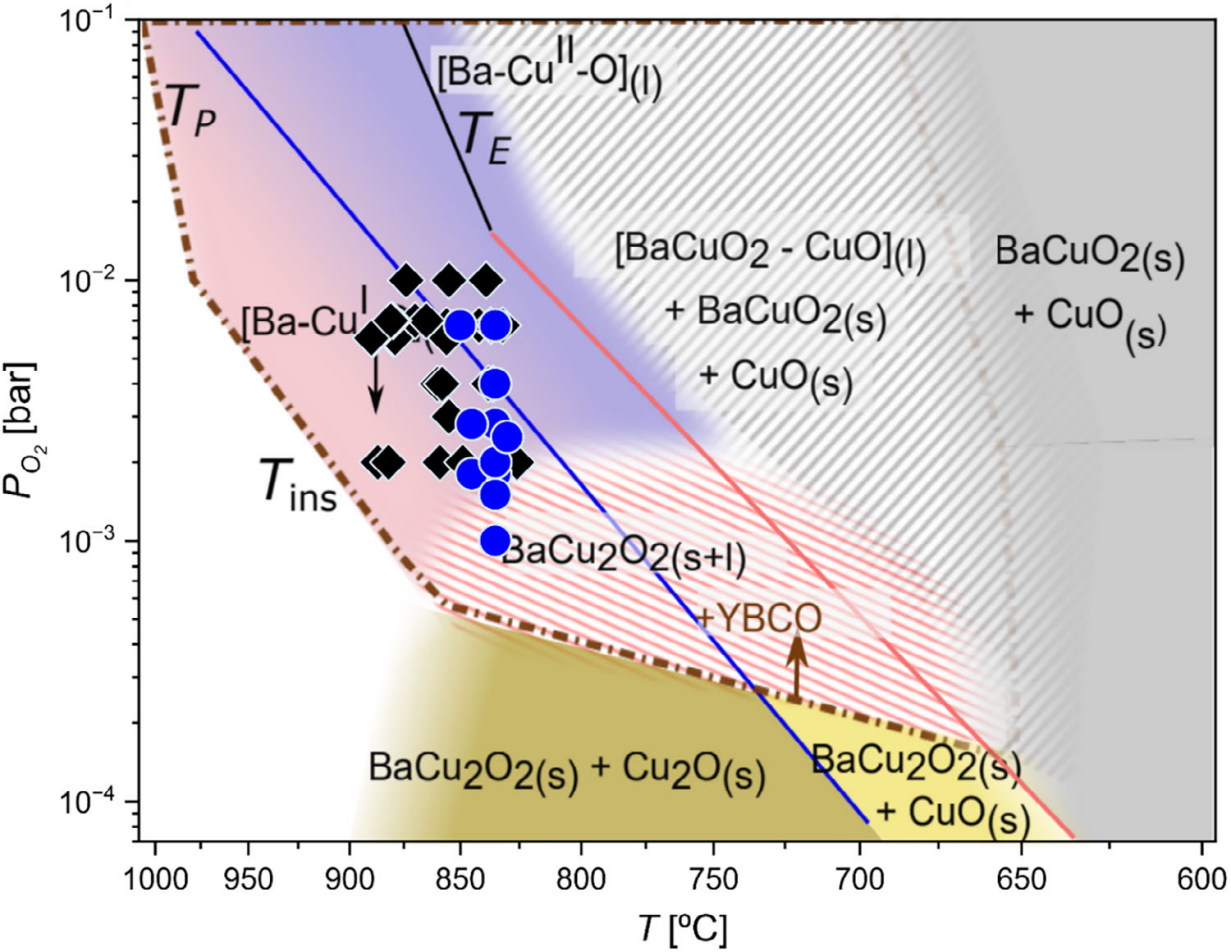
*T*‐*P_O2_* conditions relative to the growth of YBCO films through TLAG followed by in situ synchrotron XRD (black rhombi) and the YBCO films grown and analyzed ex situ (blue circles). Growth temperature is varied between 825 and 890 °C, whereas *P_O2_* is analyzed between the values of 1 mbar and 10 mbar. The coloring of the background refers to the TLAG kinetic phase diagram, adapted from.^[^ ^28^ ^]^ Shaded regions varying from blue to pink are relative to the formation of a full [Ba‐Cu^(I/II)^‐O] transient liquid with blue corresponding to Cu^2+^ and pink to Cu^+^. Striped regions indicate coexistence of solid and liquid phases. Solid lines correspond to the following phase transformations: CuO_(s)_ – Cu_2_O_(s)_ (blue line); BaCuO_2(s)_‐BaCu_2_O_2(s)_ (dark pink line); eutectic melting of BaCuO_2(s)_‐CuO_(s)_ (*T_E_*); peritectic melting of YBCO (*T_P_*); YBCO instability line (*T_ins_*). Phase transformations at lower *P_O2_* values have been previously described in.^[^ ^28^ ^]^

To comprehensively understand the parameters controlling the nucleation and growth of YBCO through TLAG from different supersaturation conditions, a wide variety of growth profiles have been tested on films of the five liquid compositions and analyzed ex situ. Several contributions to the final epitaxy must be considered to identify the tendencies correlated to liquid supersaturation determining high‐performance film properties. A total of 57 films were grown through TLAG P_O2_‐route in the temperature range between 825 and 850 °C and *P_O2_* values extended over the range 1–7 mbar, as it can be seen in **Figure** **6** (blue circles).

The contribution of the epitaxial texture (*Δω*) to the critical current density (*J_c_*) measured for all films was analyzed. We observe a comparable trend at both 5 K and 77 K (in self‐field). The films of compositions with lower copper content exhibit generally larger values of *Δω*, suggesting worsening of the texture in films of these liquid compositions, as previously evidenced by the phase evolution plots of the in situ XRD in Figure 5a–d. The elevated supersaturation intrinsic to these compositions is enhancing the mixed nucleation resulting in low epitaxy and, thus, a general drop in the values of *J_c_* for both (2:3) and (3:6) compositions.^[^ ^41^ ^]^ Conversely, the compositions richer in copper, with intrinsically lower transient liquid supersaturation, favor full c‐axis nucleation resulting in higher epitaxy and, thus, generally more elevated values of critical current density. However, despite this trend, there is a significant overlapping of the *Δω* values of all compositions around the range 0.25–0.30° in which the *J_c_* is strongly increased only in the films of high copper compositions, i.e., (3:7) and (4:11) (see Figure S4, Supporting Information for more information). Having a high‐quality film texture is fundamental for physical performance but it is not the sole requirement to achieve high critical current densities.

### 2.3 Competing Paths to YBCO Epitaxy

**Figure 7:**
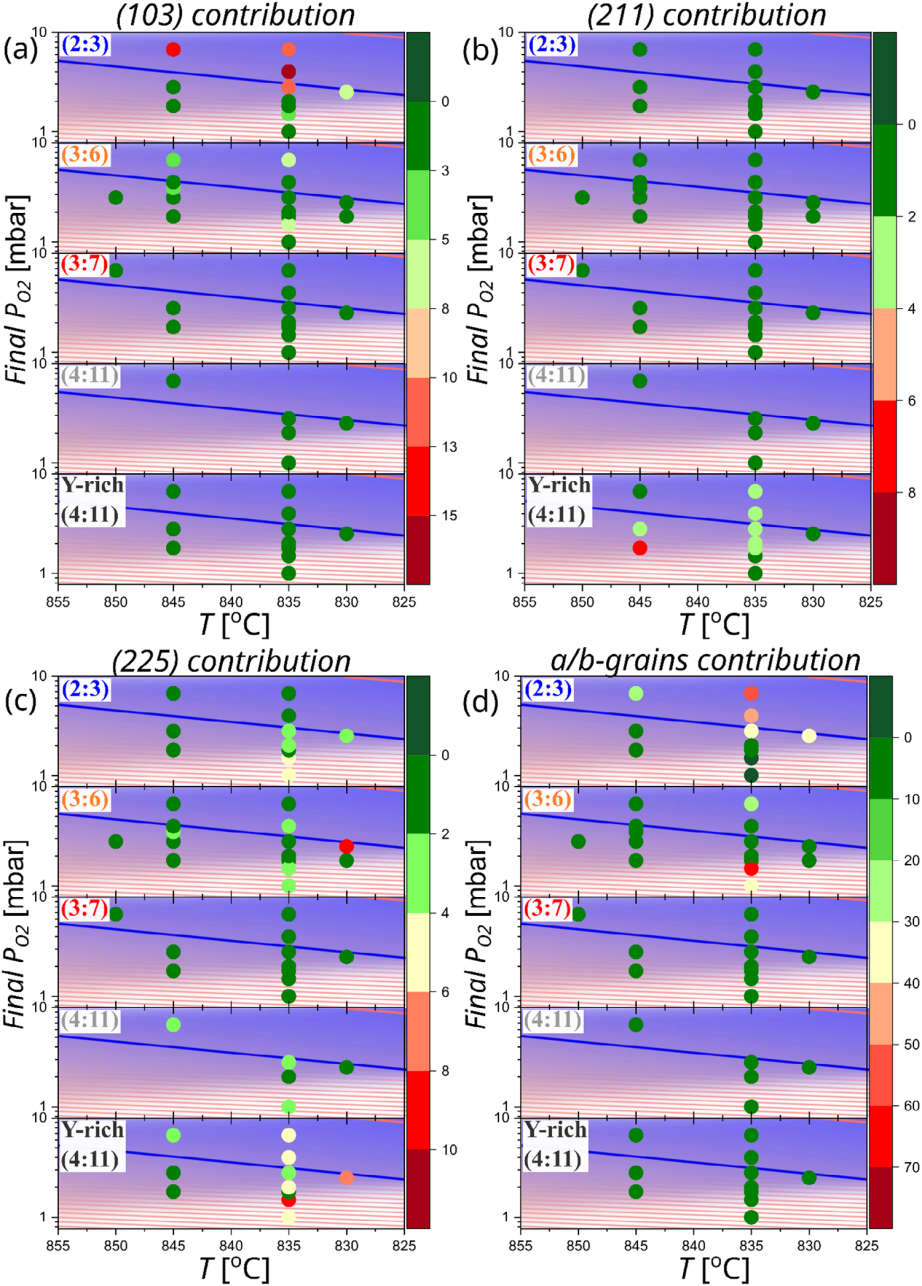
Quantification of the non‐epitaxial contributions (as estimated from the percentage of the Bragg peak ratios indicated in Equation 4 of the article). A selected Bragg peak of the non‐epitaxial contribution is normalized to the YBCO (005) Bragg peak associated to the c‐axis epitaxy of the films grown from different compositions and under different conditions. The background color maps correspond to those used on the TLAG kinetic phase diagram and the blue line corresponds to the CuO_(s)_ – Cu_2_O_(s)_ phase transformation (Figure 6), and separate plots are shown for each of the five transient liquid compositions. The points correspond to the specific *T*‐*P_O2_* conditions in which each film is grown. Contributions are a) randomly oriented YBCO estimated from (103) Bragg peak, b) (211) secondary phase estimated from the (132) Bragg peak of Y_2_BaCuO_5_ phase, c) (225) secondary phase estimated from the (211) Bragg peak of Y_2_Cu_2_O_5_ phase, and d) YBCO a/b‐grains estimated from (200) Bragg peak. For reference, a full range 2D‐XRD pattern for a film of each liquid composition is reported in Figure S5 (Supporting Information), displaying the varying intensities of each secondary phase contribution. The liquid composition is indicated in top‐left of each panel. The choice of colors for each scale is intended to visually consider a range extending from the complete absence (corresponding to a dark green) to the high presence or predominance (corresponding to a bright red) of a particular phase. For easier visual understanding, the numerical values in the scale of each plot (displayed on the right of each group of graphs) is adapted according to the maximum intensity of each non c‐axis contribution.

To identify the contributions to the drop in critical current density, we performed a detailed analysis of the ex situ 2D‐XRD patterns of all films. Integration in *χ* of the peaks relative to phases judged as a disruption to the full epitaxy allowed for a quantification of the contribution (as a percentage value of Bragg peak intensities) of the individual phase to the epitaxial moiety (Equation 4, see Experimental Section). The non‐epitaxial contributions (“non‐epi”) considered to be of major relevance toward the worsening of the epitaxial film quality are a/b‐grains and homogeneously nucleated YBCO grains (random orientation, (103) Bragg peak), and the secondary phases of Y_2_BaCuO_5_ (211) and Y_2_Cu_2_O_5_ (225). In **Figure** **7** the contribution of each one of the listed non c‐axis contribution versus the YBCO (005) peak is represented for each individual liquid composition including the background color map of the TLAG kinetic phase diagram (Figure 6), with each point indicating the specific *T*‐*P_O2_* conditions. The contributions of randomly oriented YBCO determined from the (103) Bragg peak and of a/b‐grains determined from (200) Bragg peak (Figure 7a,d), respectively, are the most relevant non c‐axis contributions in the compositions characterized by low copper content (high supersaturation), specifically important in films of (2:3) composition grown at elevated *P_O2_* in all the tested temperatures.

**Figure 8:**
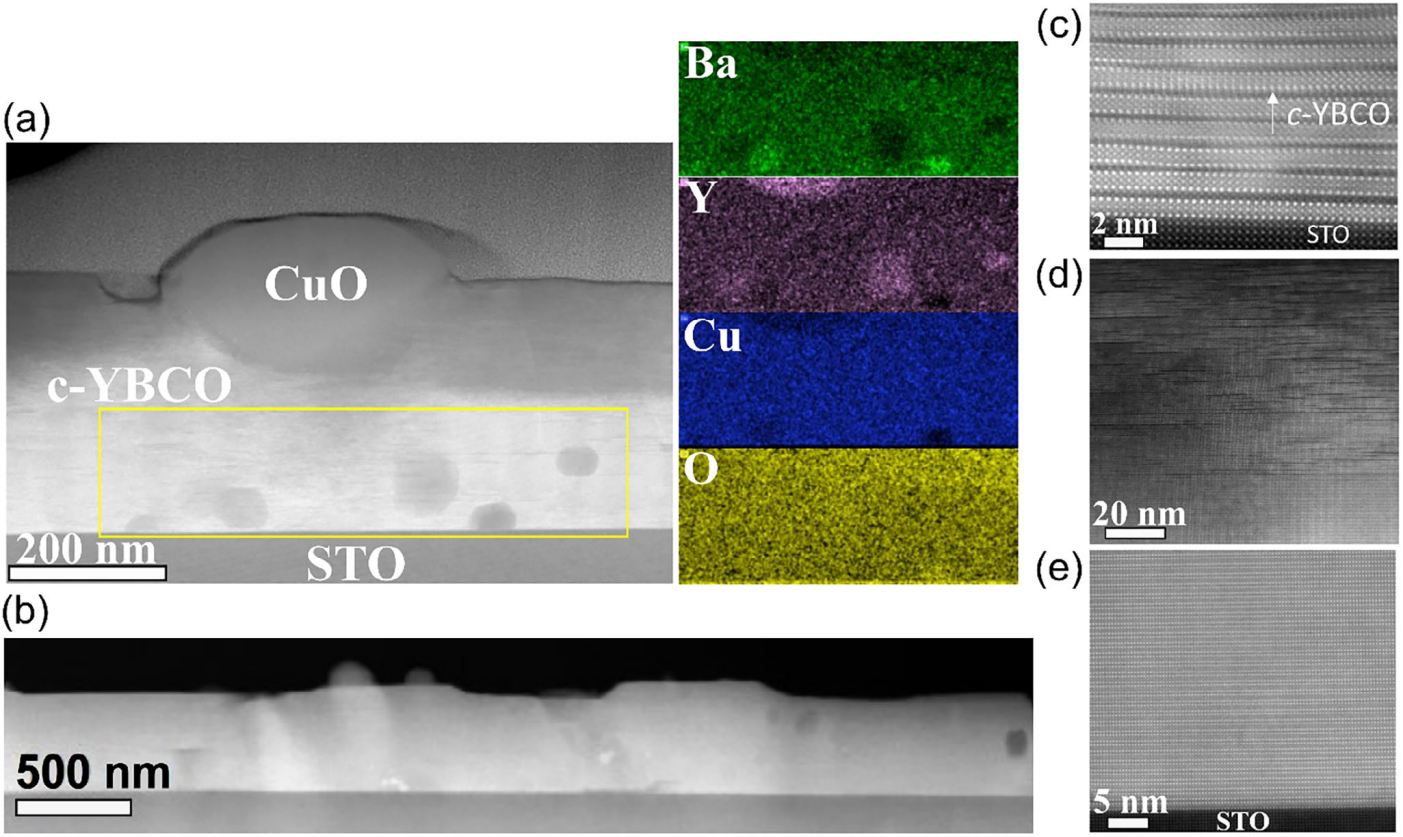
a) Cross‐sectional STEM‐HAADF image of a film of Y‐rich (4:11) composition grown through TLAG P_O2_‐route (*T* = 835 °C, *P_O2_* = 1 mbar). STEM‐EDX elemental maps are acquired on the region of the film indicated by a yellow rectangle for Ba L‐edge, Y K‐edge, Cu K‐edge, and O K‐edge, and allow for the identification of the secondary phases. b) Cross‐sectional STEM‐HAADF image of a film of (3:7) composition grown through TLAG P_O2_‐route (*T* = 835 °C, *P_O2_* = 1 mbar). c) Evidence of the formation of long (248) intergrowths, distorting the YBCO lattice. d) Atomic resolution STEM‐HAADF image displaying the growth of highly epitaxial YBCO in the Y‐rich (4:11) films. Darker area is corresponding to the substrate (STO) single crystal lattice. e) Atomic resolution STEM‐HAADF image of a (3:7) film where long (248) intergrowths are visible.

Supersaturation increases with the increase of *P_O2_*:^[^ ^41^ ^]^ for same processing conditions, the supersaturation is higher in liquid compositions with low copper content, thus favoring the emergence of a/b‐oriented YBCO and misoriented grains due to the competing homogeneous‐heterogeneous nucleation. Films of copper‐rich compositions exhibit null contributions deriving from a/b‐grains in all the tested conditions, demonstrating that the decrease in supersaturation through tuning of the transient liquid composition is successfully promoting the c‐axis growth in the region investigated. Nonetheless, the (4:11) and Y‐rich (4:11) compositions display the highest presence of (211) and (225) phases (Figure 7b,c, respectively). Evidently, Y‐rich (4:11) composition greatly favors the nucleation of (225) grains, given the large excess of copper. Nonetheless, this phase is present in the other compositions as well, with the sole exception being the eutectic one. Formation of secondary phases is presumably favored in the compositions of lower copper content due to the non‐ideal nucleation conditions for c‐axis YBCO, as explained in Section 2.2, the initial hindrance to the elimination of BaCO_3_ is an additional parameter negatively affecting the full formation of the transient liquid. Besides favoring homogeneous nucleation of YBCO, the possible inhomogeneities in the transient liquid layer will lead to competition between growth of specific secondary phase and YBCO. For what concerns the contribution of the (211) secondary phase (Figure 7b), the sole composition presenting significant formation of such crystalline phase is, again, the Y‐rich (4:11) composition in several of the tested growth conditions. Promotion of the nucleation of such phase has been previously reported in YBCO growth from Y‐rich melts, which resulted in a mixture of YBCO and (211) in the cases of melt‐textured bulk ceramic growth.^[^ ^50^, ^51^, ^52^, ^53^, ^54^, ^55^, ^56^ ^]^ The (211) phase is at times intentionally included in epitaxial YBCO samples as it was proven to enhance critical current densities due to pinning effects.^[^ ^51^, ^55^, ^57^ ^]^ Considering the high contributions of (211) and (225) secondary phases in films of high copper excess, we investigated the microstructure of films of Y‐rich (4:11) composition to ensure these phases would not be of hindrance to current percolation (**Figure** **8**). Microstructural analysis through

TEM confirms that these phases are exhibiting small grain sizes avoiding the disruption of the current percolation in the film. Moreover, if present in the appropriate size range, these may act as natural pinning centers and enhance physical properties through defect generation in the YBCO lattice, demonstrated to increase vortex pinning.^[^ ^7^, ^58^, ^59^, ^60^ ^]^

The cross‐sectional STEM‐HAADF images show that the films display homogeneous thickness of the epitaxial YBCO layer with the presence of a large grain of CuO on the film surface (Figure 8a). During epitaxial crystallization, pushing to the superficial position of the excess CuO leads to the formation of solid particles displaying a large particle coarsening. However, because these are localized at the surface, they are not influencing current percolation. For instance, the film displayed in Figure 8a has high self‐field critical current densities (*J_c_* = 23.4 MA cm^−2^ at 5K and 2.3 MA cm^−2^ at 77K). Several secondary phase grains are observed inside the (4:11) film while the (3:7) film is practically free of secondary phases. The sizes and concentrations of secondary phases depend on the film composition and thickness (Figure 8a,b; Figure S7a, Supporting Information), however these particles are not disrupting the film continuity because their size is significantly smaller than the film thickness. Through the acquisition of STEM‐EDX elemental maps acquired for Ba L‐edge, Y K‐edge, Cu K‐edge, and O K‐edge, the identification of these secondary phases is possible (Figure 8a; Figure S6, Supporting Information). As expected, due to the liquid composition of the (4:11) film, the secondary phases are predominantly yttrium‐ and copper‐rich, identifiable with the mixed oxide Y_2_Cu_2_O_5_. The additional minor presence of grains composed by barium and copper suggest the formation of the mixed oxide of these elements. Epitaxial YBCO films grown through TLAG are characterized by (Y248) intergrowths (Figure 8c,e), and in the Y‐rich (4:11) film, they are majorly present in the upper portion of the film while in the (3:7) film they appear at the substrate interface. However, the growth of highly epitaxial YBCO is obtained, as displayed in the atomic‐resolution STEM‐HAADF images of the interfaces (Figure 8d,e; Figure S7a,b, Supporting Information). Further detailed considerations on the defect landscape resulting from TLAG are currently under study but are out of the scope of this work.

An additional correlation with the morphological analysis of film surface is presented in Figure S8 (Supporting Information), where exemplary features of the presence or absence of the secondary phases presented in Figure 7 are identified. In general, the compositions richer in copper present a smooth surface with the excess of copper found as superficial precipitates. Major features considered to be hindering current percolation are most commonly visible in the films of lower copper content liquids, which are exhibiting granular morphology and dewetting phenomena, the latter mainly found in proximity of large grains of secondary phases or misoriented YBCO.

**Figure 9:**
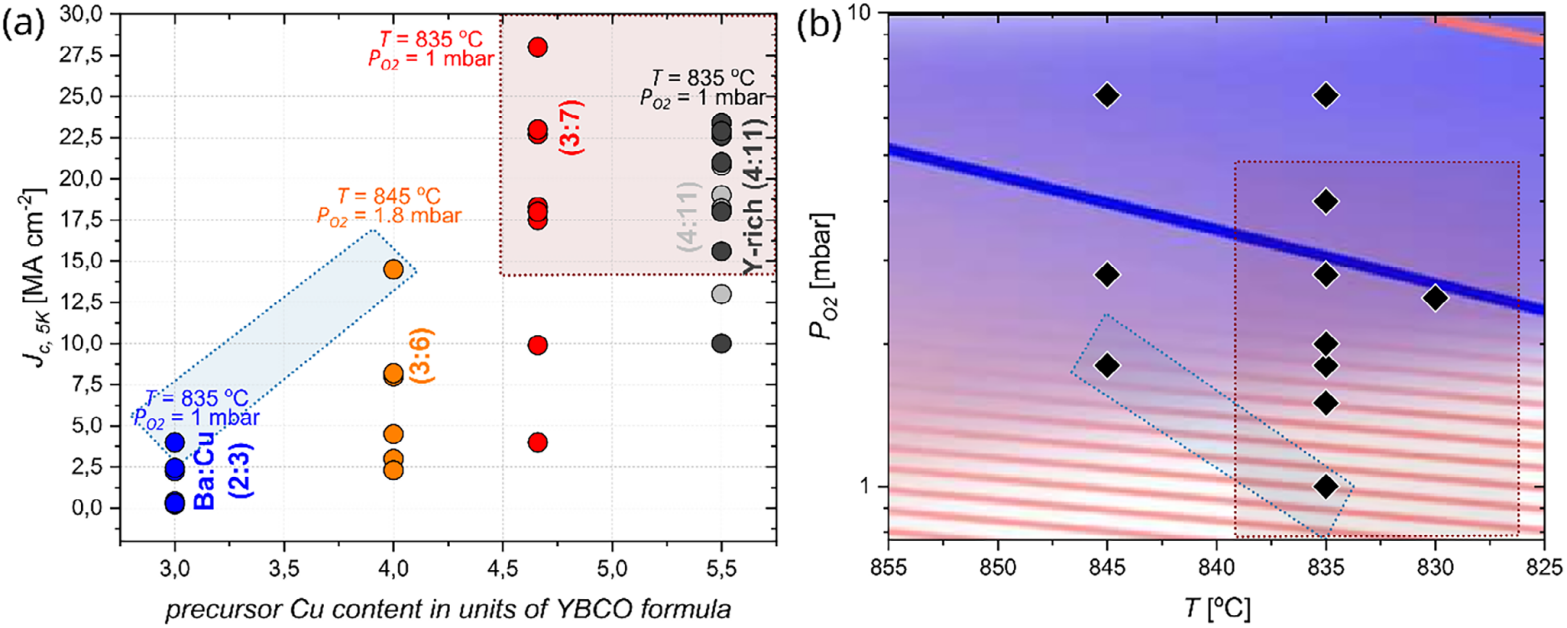
a) Values of critical current density (*J_c_*) measured at 5 K in self‐field as a function of the copper content in each liquid composition, relative to films grown through TLAG P_O2_‐route. Shaded areas are relative to the best performing films of compositions with low copper content (blue shading) and high copper content (red shading). Lower *J_c_* values correspond to films grown under non‐optimal conditions. *T*‐*P_O2_* conditions for highest performing film of each composition is reported as a label next to the relative symbol (with the same color as the symbols of each composition). b) Identification of high‐performance window of *T*‐*P_O2_* conditions resulting from enhancement of epitaxy for films of low copper content (blue shading) and copper excess (red shading). The blue line corresponds to the CuO_(s)_ – Cu_2_O_(s)_ phase transformation, as indicated in Figure 6.

An overview of the correlation of liquid composition with the critical current density is displayed in **Figure** **9**a, where the self‐field critical current density values at 5 K are plotted as a function of the copper content in each liquid composition when samples are processed under different conditions. (2:3) and (3:6) composition films exhibit maximum *J_c_* values of 4 and 14.5 MA cm^−2^, respectively. In contrast, besides the higher maximum *J_c_* values, films deriving from the eutectic liquid composition (3:7) and the ones with higher copper excess (4:11) display critical current densities in a range of more elevated values, with the lowest‐performing cases (corresponding to non‐optimal growth conditions) coinciding with the leading ones from the compositions with less copper. As similarly observed for the analysis of secondary phases, when relating the *T*‐*P_O2_* conditions to the enhancement of *J_c_* values (Figure 9b), we can identify a wide window of processing conditions where values of *J_c_* are ranging between 15 MA cm^−2^ and 28 MA cm^−2^ (5 K in self‐field) for compositions with high copper content (red shaded region). These films grown under the optimal conditions and having the highest *J_c_* values at 5 K also displayed high superconducting performance at 77 K and self‐field (2.7 MA cm^−2^), thus demonstrating that they reached competitive values as compared to most of the commercial coated conductors grown through other methodologies.^[^ ^7^ ^]^ Moreover, we achieved comparable film microstructure and superconducting performance when doubling the film thickness (Figures S7 and S9, Supporting Information), demonstrating true feasibility of TLAG for industrial implementation, where increased film thickness is a fundamental requirement.

Contrarily, compositions with lower copper content display a reduced window of *T*‐*P_O2_* conditions corresponding to improved physical properties. The *J_c_* values range of films with composition (2:3) and (3:6), here, between 4 MA cm^−2^ and 14 MA cm^−2^ (5 K in self‐field), is a further confirmation of the importance of a correct control of the transient liquid supersaturation through *T* and *P_O2_* parameters toward the fabrication of high‐performance YBCO films. The absence of a direct correlation between the prevailing presence of secondary phases of (211) and (225) in films of Y‐rich (4:11) liquid composition and an expected decrease in the physical properties finds an explanation in the TEM images in Figure 8a. Despite the formation of several grains of the secondary phases, the superconducting properties of the films of this composition display high‐performance given the reduced size of these grains which are not an obstacle to current percolation or present no major disruption of the highly epitaxial grown YBCO.

The correlation between transient liquid supersaturation and epitaxial YBCO nucleation is a multi‐parameter problem: several factors must be controlled to enhance the final physical performance of the superconducting films. A tight control of the processing parameters for each liquid composition is required to tune the supersaturation and then avoid a decrease in the physical properties. The quality of the YBCO film epitaxy is a fundamental parameter to be controlled, and low values of *Δω* may result in increased physical properties of the film, if other parameters are not affecting the outcome. However, it is evident that for compositions of lower copper content, even when *Δω* values were reduced, no improvement to the performance of the films could be achieved, suggesting the important role of other parameters. In the tested processing conditions, the high supersaturation of the melts deriving from the (2:3) and (3:6) compositions are greatly favoring the homogeneous nucleation of YBCO, with strong presence of non‐epi contributions. The highly defective surface morphologies are characteristic of poor epitaxy and are additionally displaying dewetting phenomena, contributing to current percolation impediment and to a general drop in the physical properties of such films. To overcome such complications, higher processing temperatures are believed to be beneficial for (2:3) and (3:6) composition films. However, using copper‐rich liquid compositions, a general lowering of the supersaturation of the [Ba‐Cu‐O] transient liquid is obtained, directly correlated to the attainment of highly epitaxial films at lower temperature processing conditions, where the compositions of lower copper content display mixed nucleation. Additionally, considering that one of the future objectives is to facilitate the industrial application of the TLAG‐CSD approach, higher processing temperatures are undesirable, especially considering the general implementation of energetically efficient processes for CCs production. For this reason, films deriving from the eutectic and the copper‐excess liquid compositions are more appealing toward the use in the industrial framework, given the wide high epitaxy window found at lower temperatures. The lower supersaturation characteristic of the transient liquids of these compositions are determining excellent heterogeneous nucleation in a wide region of *P_O2_* values together with the possibility of maintaining a reduced growth temperature. High‐performance properties are obtained in several *T*‐*P_O2_* conditions in films of (3:7), (4:11) and Y‐rich (4:11) compositions, demonstrating that a careful control of the transient liquid supersaturation contributes to the achievement of full c‐axis YBCO nucleation with negligible presence of secondary phases and optimal surface morphology. Moreover, despite the films of Y‐rich (4:11) composition experience the formation of significant amounts of (211) and (225) secondary phases, if these can be kept in small sizes high *J_c_* values are obtained, as we could demonstrate in this study.

## 3 Conclusion

We presented a detailed analysis of the multiple parameters correlating the transient liquid supersaturation, intrinsic to the initially defined film composition, and the epitaxial nucleation of YBCO in TLAG. Few works previously reported the solubility of Y in [Ba‐Cu‐O] melts of different compositions and were all performed in processing conditions far from the working window of TLAG. Therefore, the results presented in this manuscript are believed to be of great interest for the community, given the possibility of a straightforward control of the transient liquid supersaturation through the simple variation of its copper content. Using copper‐rich compositions, specifically the eutectic (3:7) composition and the copper‐excess (4:11) and Y‐rich (4:11) compositions, important improvement of the epitaxy was achieved at lower temperatures compared to the compositions with lower copper content (namely, (2:3) and (3:6) compositions). The reason is thought to lie in the higher solubility of Y in transient liquid of higher copper content, as previously suggested by the studies in MTG, LPE, and HPLE. When the equilibrium solubility of a RE in a melt increases, its supersaturation decreases, thus if the same processing conditions are selected for several liquid compositions, the copper excess will promote the lowering of this parameter. Decreased supersaturation conditions are demonstrated to be linked to a promotion of the c‐axis nucleation, necessary for the resulting YBCO films to display high superconducting performance. The in‐depth analysis of the kinetic growth process of YBCO through TLAG performed through in situ XRD experiments revealed vital information on the evolution of the phases, unattainable through other ex situ techniques. Highly epitaxial YBCO was obtained through in situ synchrotron XRD experiments, and additional knowledge regarding the kinetic mechanism of TLAG was generated. The use of fast acquisition during the pressure jump, besides allowing for a more accurate tracing of the phase evolution in this step of the process, led to the determination of unprecedented growth rates. This is a clear indication of the great industrial interest of TLAG, which surpasses the growth rates presented by the conventional CCs fabrication methods by several orders of magnitude while ensuring YBCO films of high‐performance, with achieved critical current density values up to 2.7 MA cm^−2^ at 77 K and 20 MA cm^−2^ at 5 K at self‐fields, i.e., in the same range of most of the existing coated conductor manufacturing approaches showing high performance. We believe this work is a further demonstration of the interest of TLAG‐CSD as a novel methodology with a high potential for coated conductor fabrication at the industrial scale.

## 4 Experimental Section

### Precursor Film Preparation

Preparation of the metal propionate powder precursors and the YBCO precursor solution preparation procedure and optimization are described, respectively in^[^ ^35^ ^]^ and.^[^ ^34^ ^]^ In this work, chemical solutions with (Ba:Cu) molar ratios of (2:3), (3:6), (3:7) and (4:11) are employed to obtain films with initial composition corresponding to YBa_2_Cu_3_O_7‐δ_, YBa_2_Cu_4_O_7‐δ_, YBa_2_Cu_4.66_O_7‐δ_, and YBa_2_Cu_5.5_O_7‐δ_. For clarification, these result in final YBCO films which are a mixture of the stoichiometric YBa_2_Cu_3_O_7‐δ_ and CuO (when copper‐excess chemical compositions are employed).^[^ ^34^ ^]^ Films deriving from chemical solutions of the four transient liquid compositions were obtained by depositing each precursor solution via spin coating in a ISO7 clean room at 10% humidity at a spinning rate of 6000 rpm on 5×5 mm^2^ single‐crystal (001) SrTiO_3_ (STO) substrates (CrysTech GmbH). Before deposition, substrates undergo an annealing process at 900 °C for 5 h to obtain flat‐terraced surfaces. The pyrolysis was performed by heating the samples in a tubular furnace in humid oxygen up to 500 °C. YBCO nanocrystalline precursor films with a thickness of ≈800 nm independent of the composition are obtained through a multideposition process, thus repeating the steps of deposition and pyrolysis twice. This process yields nanocrystalline films composed by Y_2_O_3_, BaCO_3_ and CuO with the optimal microstructural features for the ulterior TLAG process.^[^ ^34^ ^]^

### Growth of YBCO Films through TLAG P_O2_‐Route

Growth of the YBCO thin films through TLAG was performed using the previously described P_O2_‐route.^[^ ^27^, ^28^, ^34^, ^35^ ^]^ The experiments were carried out in a tubular furnace equipped with a vacuum system that enables rapid switching (time range of a second) from a high vacuum to a reduced vacuum. The samples are heated at low‐*P_O2_* (*P_O2_* = 10^−5^ bar), with an average heating rate of 1 °C s^−1^ to the desired temperature. In this system *P_O2_* = *P_TOT_*, with the desired values achieved through the inlet of high‐purity O_2_ (≥ 99.995%) and regulation through needle‐valves. In this step of the process, the reaction between BaCO_3_ and CuO leads to the formation of the solid phases of BaCu_2_O_2_ and Cu_2_O, precursors of the [Ba‐Cu^(I/II)^‐O] transient liquid. This heating profile was demonstrated to yield films with optimal precursor phases for the formation of the transient liquid. A rapid variation of *P_O2_* (namely, pressure jump) allows for the formation of the transient liquid and the following YBCO crystallization. Following TLAG, the YBCO films undergo an oxygenation process to ensure the tetragonal to orthorhombic YBCO phase transition. The samples are heated in a tubular furnace (*P_TOT_* = 1 bar), with a heating rate of 10 °C min^−1^ to 450 °C, followed by a dwell of 210 min and afterwards cooling to room temperature.

### In Situ Analysis of X‐Ray Diffraction (XRD) with Synchrotron Radiation

In situ XRD was performed at the DiffAbs Beamline of SOLEIL synchrotron and at NCD‐SWEET Beamline of ALBA synchrotron. Beam energy in both facilities was 18 keV and fast 2D detectors were used ensuring the acquisition of all precursor crystalline phases of interest in a single 2D image. Acquiring rate from 1 s frame^−1^ down to 9 ms frame^−1^ were used depending on the specific part of the process. An XRD furnace from Anton Paar (model DHS1100) equipped with a graphite dome was used as a heating stage and a homemade installation enabling the P‐route TLAG process was developed. Correct *P_O2_* is reached by introduction of high‐purity (< 10 ppm) synthetic air in the system, considering *P_TOT_* = 1/5 *P_O2_*. In the experiments described in this article, the low‐*P_O2_* heating was conducted at *P_TOT_* = 0.05 mbar and *P_O2_* = 0.01 mbar. Further details from the installations will be described elsewhere.

### Ex Situ Characterization Techniques

XRD was performed on a Bruker D8 Discover system (Cu Kα, X‐ray energy = 8.049 keV) equipped with a Lynxeye XE‐T energy‐dispersive one‐dimensional (1D) detector, measuring in grazing incidence (GI) geometry to characterize structure and phase composition, and on a Bruker‐AXS D8 Advance diffractometer (Cu Kα equipped with general area detector diffraction system (GADDS)) used to characterize structure and phase composition of the grown YBCO layers. Analysis of the contribution of each secondary phase or non c‐axis oriented YBCO (defined as “non‐epi”) versus the film c‐axis epitaxy (defined as “epi”) is conducted by fitting the peaks relative to all secondary phases (using the (132) Bragg peak of Y_2_BaCuO_5_ for the (211) secondary phase, while the (211) Bragg peak of Y_2_Cu_2_O_5_ for the (225) secondary phase) and YBCO (005), (103) and (200) reflections in the integrated 2D‐XRD patterns. The parameter employed to quantify the non c‐axis contributions (as a percentage versus the maximum observed contribution) is expressed by Equation (4):

(4) $$non\;-\;epi\;contribution=\frac{PeakArea_{\left(non-epi\right)}}{\left[PeakArea_{\left(non-epi\right)}+PeakArea_{\left(epi\right)}\right]}\;\;\cdot 100$$

Surface morphology was analyzed with scanning electron microscopy (SEM) and energy‐dispersive X‐ray (EDX) spectroscopy with a QUANTA FEI 200 FEG‐ESEM.

For microstructural studies, cross‐sectional TEM lamellae were prepared by Thermo Scientific Helios 5 DualBeam focused ion beam (FIB). Atomic‐resolution high‐angle annular dark field scanning transmission electron microscopy (HAADF‐STEM) images were acquired using a ThermoFisher Scientific Spectra 300 STEM with double‐aberration correction, operated at 300 kV. Elemental compositional maps of energy dispersive X‐ray spectroscopy (EDX) were acquired using a Super‐X EDS detector.

The self‐field critical current density (*J_c_*) values were obtained from out‐of‐plane inductive measurements in a commercial Quantum Design MPMS XL SQUID DC magnetometer equipped with a 7 T magnet. *J_c_* self‐field (at 5 K and 77 K) was deduced from the remanent magnetization by applying the Bean critical state model for a thin disk.

## Conflict of Interest

The authors declare no conflict of interest.

## Author Contributions

L.S., X.O., T.P., conceived the idea and designed the growth experiments. L.S., D.G., D.S.‐R., J.A., V.F., E.P., J.F., and T.P. performed the in situ XRD experiments under the guidance of E.S. (at NCD‐SWEET Beamline, ALBA synchrotron) and of C.M. (at DiffAbs Beamline, SOLEIL synchrotron). L.S. analyzed related data and prepared the relative graphs, following discussion with J.F., X.O. and T.P., L.Sol. contributed to the discussions and estimations of the RE supersaturation in the liquid. K.G. performed the TEM microstructural studies and analyzed the data. T.P. and X.O. oversaw the project progress. L.S. wrote the manuscript with participation from all authors in the revision process. All authors have given approval to the final version of the manuscript.

## Supporting information



Supporting Information

## Data Availability

The data that support the findings of this study are available from the corresponding author upon reasonable request.
