# Supplementary material for: Role of Liquid Composition in the Transient Liquid Assisted Growth of Superconducting YBa2Cu3O7‐δ Films
Source: Adv Mater. 2025 Sep 9;38(51):e10660. doi: 10.1002/adma.202510660 (PMC13568875; doi:10.1002/adma.202510660)
Supplement: Supplementary file 1 [file ADMA-38-e10660-s001.docx]

Supporting Information

Role of Liquid Composition in the Transient Liquid Assisted Growth of Superconducting YBa_2_Cu_3_O_7-δ_ Films

Lavinia Saltarelli, Diana García, Laia Soler, Elzbieta Pach, Kapil Gupta, Daniel Sanchez-Rodriguez, Jordi Aguilar, Víctor Fuentes, Eduardo Solano, Cristian Mocuta, Jordi Farjas, Xavier Obradors*, Teresa Puig

**Table of Contents**

**Section I. *Y supersaturation in [Ba-Cu-O] melts***…………………………………….….page 3

**Section II. *Effect of Cu content on elimination of BaCO_3_***……………………………...page 3

**Section III. *Influence of film composition on epitaxial YBCO nucleation***………….....page 6

**References**………………………………….…………………………………...............page 11

**Section I. *Y supersaturation in [Ba-Cu-O] melts***

**Figure S1** - Extrapolation of the value of *C_δ_* = 10 %, through the extension of the liquidus line of Y_2_O_3_ + liquid thermodynamic stability. Formation of YBa_2_Cu_3_O_x_ is corresponding to the complete dissolution of all the Y present (17%). Generally, participation of the full amount of this element in the melt is an extremely rare phenomenon to occur, given the formation of secondary phases containing Y. Phases are labelled as follows: 123 -YBa_2_Cu_3_O_x_; 211 - Y_2_BaCuO_5_. Adapted from ^[1]^.

**Section II. *Effect of Cu content on elimination of BaCO_3_***

Here, an analysis of the effects of compositional variations on the reaction between BaCO_3_ and CuO to form the mixed oxide BaCu_2_O_2_, occurring during the low-P_O2_ heating, is studied.

When varying the copper content in the films, we observe significant differences in the microstructural features after pyrolysis,^[2]^ as reflected in the XRD patterns acquired following this first heat treatment displayed in Figure S2 and Figure S3a. A correlation between the crystallinity exhibited by the phases in each film and the liquid composition can be identified: films with lower copper content, specifically the stoichiometric (2:3) composition and the one with lowest copper excess, (3:6) composition, display a general increased crystallinity, significantly pronounced for the BaCO_3_ reflections. Contrarily, the compositions with copper excess, (3:7) and Y-rich (4:11) compositions, are characterized by a reduced crystalline aspect. We believe an explanation for this feature may be found in the intrinsic variation of the copper content which shifts BaCO_3_ nucleation to higher temperatures.^[3]^ Given the same thermal profile is employed for all films in pyrolysis, the result is that films with higher copper content display an increased amorphous character respect to the ones with low copper content, which have crystallized sufficiently throughout the duration of the thermal treatment.

**Figure S2** - XRD analysis of nanocrystalline precursor films (acquired with X-Ray energy of 8.049 keV) of the four compositions after pyrolysis. From left to right, we start from the stoichiometric (2:3) composition and increase the copper content up to the Y-rich (4:11) composition.

When employing these films in TLAG, a tendency between microstructural features after pyrolysis and BaCO_3_ elimination can be observed (Figure S3b). The advantageous possibility of the P_O2_-route of decoupling the limiting step of the process to the formation of the transient liquid, and successive YBCO nucleation, allows us to analyze in more detail the effects of film composition on BaCO_3_ elimination. Following the low-P_O2_ heating, the contribution of BaCO_3_ phases is especially elevated for (2:3) and (3:6) compositions. This may be deriving from a combination of two effects: the initially elevated crystalline character of BaCO_3_ phases after pyrolysis implies a higher thermal stability, hindering the complete removal of these phases at the selected processing conditions. Additionally and most importantly, the lower copper contents of these compositions denotes a higher (Ba:Cu) ratio compared to the one of the films with copper excess, producing an impairment in the catalytic effect of CuO towards its reaction with BaCO_3_ to form BaCu_2_O_2_. The chosen processing temperature seems, thus, not to be sufficient for BaCO_3_ full elimination, suggesting both these compositions require the implementation of higher temperature. Nonetheless, considering both the ideal reduction of the processing temperatures for HTS fabrication processes and the likelihood of coarsening of solid phases directly linked to an excessive temperature, the use of compositions with a copper excess seems to be beneficial. In fact, (3:7) composition and Y-rich (4:11) composition are showing a null contribution deriving from both BaCO_3_ phases, with the successful formation of the solid precursor phases for the transient liquid formation, namely BaCu_2_O_2_ and Cu_2_O. A combination of the catalytic effect of CuO, here in large excess, and the higher amorphous character of the initial nanocrystalline films are presumably facilitating complete BaCO_3_ removal in the case of films of these compositions, suggesting the achievement of optimal microstructural and compositional conditions for TLAG.

**Figure S3** – XRD acquired (a) prior and (b) following the low-pressure heating (*T* = 850 ºC s^−1^, heating rate *dT / dt* = 4.5 ºC s^−1^ at a constant pressure of *P_TOT,base_* = 0.05 mbar, corresponding to *P_O2,base_* = 0.01 mbar) for films of the four compositions. The profiles were acquired at SOLEIL synchrotron with X-Ray energy of 18 keV. The presence of BaCO_3_ with higher crystallinity in films of lower copper content ((2:3) composition shown in blue and (3:6) composition shown in orange) is believed to be hindering its complete elimination in these films, evidenced by the presence of peaks relative to BaCO_3_ in the plots in (b). Films of higher copper content, (3:7) shown in red and (4:11) in grey, display, contrarily, low contribution of BaCO_3_ prior to the heating, suggesting a more amorphous nature of this specific phase in these compositions. Its complete elimination and formation of the mixed oxide of BaCu_2_O_2_ in compositions with higher copper content (right panel of (b)), is confirming these films are successfully resulting in the desired solid precursor phases (BaCu_2_O_2_ and Cu_2_O) for the transient liquid formation in the successive step of TLAG.

**Section III. *Influence of film composition on epitaxial YBCO nucleation***

**Figure S4** – Critical current density (*J_c_*) values measured at (a) 5 K and (b) 77 K in self-field as a function of the *Δω* obtained from the rocking curves of the films of the five liquid compositions grown in all the tested processing conditions in TLAG P_O2_-route.

**Figure S5 -** Full range 2D-XRD patterns for films of the five liquid compositions analyzed in Figure 7 following the growth through TLAG at *T* = 835 °C and *P_O2_* = 1 mbar. Peak identification according to the *2θ* position is specified at the top of the graph, and the liquid composition relative to each 2D-XRD pattern is indicated inside the corresponding panel. It is worth noting the low intensity of the secondary phases respect to the epitaxial contribution deriving from the peak of YBCO (005).

**Figure S6 -** (a) Full range 2D-XRD of the film of Y-rich (4:11) composition displayed in the TEM images in Figure 8. Contributions from secondary phases of (211) and (225) are clearly evident, despite showing a significantly lower intensity compared to the epitaxial YBCO (005) peak. (b) STEM-EDX elemental maps are acquired for Ba L-edge, Y K-edge, Cu K-edge, and O K-edge, and, together with (c) EDX quantification from the region in the red square, allow for the identification of the secondary phases, in this case a grain of (225).

**Figure S7** - (a) Low magnification cross-sectional STEM-HAADF image of a four-layered (3:7) composition YBCO film grown through TLAG P_O2_-route. Secondary phases labels are placed in proximity of the corresponding grain. (b) HR-TEM image displaying the defect-free c-axis oriented YBCO grown on the STO (001) substrate. (c) Evidence of the presence of 248 intergrowths close to the substrate-film interface, showing a distortion of the lattice.

**Figure S8** – SEM images used for the analysis of surface morphological features of films of the five liquid compositions grown through TLAG P_O2_-route, represented on a *T*-*P_O2_* diagram with corresponding processing conditions.

**Figure S9 -** (a) SEM image displaying the surface morphology of a four-layered (3:7) composition YBCO film of final thickness 800 nm. Flat surface is observed, with the excess of copper deriving from the intrinsic copper-excess film composition found in form of precipitates at the surface. This type of surface morphology is considered ideal for films of (3:7) composition. (b) Comparison of curves of *J_c_* as a function of temperature for two films of different thickness (specified in legend) grown in TLAG P_O2_-route, demonstrating that the physical performance of the final YBCO is maintained when doubling the thickness from 400 nm to 800 nm.

**References**

[1]	T. Aselage, K. Keefer, *J. Mater. Res.* **1988**, 3, 1279.

[2]	L. Saltarelli, K. Gupta, S. Rasi, A. Kethamkuzhi, A. Queraltó, D. Garcia, J. Gutierrez, J. Farjas, P. Roura-Grabulosa, S. Ricart, X. Obradors, T. Puig, *ACS Appl. Mater. and Interfaces* **2022**, 14, 48582.

[3]	S. Rasi, L. Soler, J. Jareño, J. Banchewski, R. Guzman, C. Mocuta, M. Kreuzer, S. Ricart, P. Roura-Grabulosa, J. Farjas, X. Obradors, T. Puig, *Journal of Physical Chemistry C* **2020**, 124, 15574.
